# Impact of Adult Day Service Center Closures in the Time of COVID-19

**DOI:** 10.1093/geroni/igaa057.3472

**Published:** 2020-12-16

**Authors:** Paayal Vora, Lydia Missaelides, Chau Trinh-Shevrin, Tina Sadarangani

**Affiliations:** 1 Northeast Ohio Medical University (NEOMED), Marion, Ohio, United States; 2 California Association for Adult Day Services, Sacramento, California, United States; 3 NYU School of Medicine, New York, New York, United States; 4 New York University, New York, New York, United States

## Abstract

Adult Day Service Centers (ADCs) are a form of community-based long-term care that address frail older adults’ health and social needs. Due to their congregate nature and participants’ compromised health, many ADCs have been forced to temporarily shutter during the COVID-19 pandemic. It is unknown how closures have impacted service delivery at ADCs. Guided by the Resiliency Framework, we (1) explore methods employed by ADCs during the pandemic to meet participant/caregiver needs and (2) determine how/whether these methods have mitigated the negative effects of ADC closures on participants and their caregivers. Both virtual focus groups and one-on-one semi-structured qualitative interviews were conducted with ADC staff members (n=20) across the United States. Preliminary results showed precipitous declines in physical, cognitive, and mental health of participants, as well as increased caregiver strain, particularly among dementia caregivers. However, ADCs found creative solutions to care for participants despite restrictions, creating, in their words, “centers without walls.” Staff developed virtual programs (e.g. support groups, music and exercise therapy) and conducted “door-step” visits to support productive engagement and combat loneliness. Telehealth supported care coordination and identification of emergent clinical problems. Results suggest that despite innumerable COVID-19-related challenges, ADCs demonstrated resilience and creativity in managing participants’ needs, fostered a sense of purpose, and provided caregiver respite. Further research on the effectiveness of remote programming and advocacy for sustainable reimbursement by payers is needed to ensure that ADCs can continue to provide older adults and their families with meaningful support as the pandemic wears on.

